# Hyaluronic Acid Injection Techniques for Lip Augmentation—Comparison of Linear, Retrograde, and Microdeposit Approaches: A Systematic Review

**DOI:** 10.1093/asjof/ojag059

**Published:** 2026-04-02

**Authors:** Victor Rene Miranda Cerna

## Abstract

Hyaluronic acid (HA) is widely used for lip augmentation; however, the safety and performance of injection techniques and instruments (needle vs cannula) remain unclear. The aim of this study was to evaluate the safety of HA injection techniques for lip augmentation and to assess patient satisfaction, injected volume, and associations with technique, instrument, and HA product. A systematic review was conducted accordance with PRISMA 2020 and registered in PROSPERO (CRD420251123328). PubMed/MEDLINE, Embase, Cochrane Library, LILACS, SciELO, and Scopus were searched from January 1, 2010, to August 16, 2025, for studies involving adults undergoing HA lip augmentation. Eligible designs included randomized and nonrandomized clinical trials, cohort studies, and systematic reviews. The primary outcome was safety, defined by type and timing of complications. Secondary outcomes included patient satisfaction, injected volume, and associations with technique, instrument, and HA product. Risk of bias was assessed using RoB 2 and ROBINS-I, and findings were synthesized narratively. Sixteen studies including 3692 patients were analyzed. Most protocols used 1 to 2 mL per session, commonly employing linear retrograde techniques with serial puncture, fan patterns, or microdeposits. Adverse events were mild and transient, including edema, ecchymosis, tenderness, and nodularity, with no reported cases of vascular occlusion or vision-threatening events. Needle-only and combined needle plus cannula approaches showed comparable safety profiles. Patient satisfaction was high in most patients. HA lip augmentation appears effective and generally safe when performed by experienced injectors. Current evidence does not support a single superior technique, instrument, or HA formulation. Standardized studies are needed to define best-practice protocols.

**Level of Evidence: 3 (Risk)**  
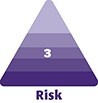

Lip augmentation with hyaluronic acid (HA) has become one of the most frequently performed minimally invasive procedures in aesthetic medicine, emphasizing the lips’ critical role in facial expression, communication, and perception of attractiveness and youthfulness.^1-6^ HA-based fillers are the material of choice for this purpose because of their excellent biocompatibility, hydrophilic properties, reversibility with hyaluronidase, and favorable safety profile.^1-6^ Advances in cross-linking technology and rheological diversity have further enabled individualized treatments according to each patient's anatomy and desired outcome.^1,3-6^

Several injection techniques have been developed to optimize precision, efficacy, and safety in lip enhancement procedures. The linear/threading technique involves continuous retrograde placement of the filler to achieve uniform volume and definition. The retrograde/fanning technique allows broad coverage from a single-entry point, minimizing punctures and potential bruising.^2,5-13^ The microdeposit technique consists of delivering small aliquots at specific points to refine contour, projection, and lip texture and is frequently combined with serial puncture or cross-hatching patterns.^2,4-9,11-13^ These approaches are often combined within the same treatment session and may be performed with a needle alone or with a combination of needle and cannula, depending on the treated subunit and the desired degree of precision and tissue support.^2,5-7,9,11-14^

Despite their widespread use, the comparative evidence on these techniques remains limited, heterogeneous, and sometimes contradictory. Studies differ in injection depth and plane, choice of needle vs cannula, filler rheology, treated lip segments, follow-up duration, and outcomes measures, which complicates standardization and hinders clear conclusions regarding the relative safety or efficacy of each approach.^2-11^ Furthermore, although most complications are mild and transient—such as edema, bruising, nodularity, or local discomfort—rare but clinically relevant adverse events, including foreign-body granulomas, angioedema, and vascular compromise with potential necrosis, have been reported in association with lip fillers, underscoring the importance of precise, anatomically informed techniques.^1-3,5,6^

Imaging tools, particularly high-resolution ultrasound, have recently improved understanding of lip anatomy, vascular patterns, and filler behavior in vivo and have been proposed both for pretreatment planning and for immediate postprocedure verification of filler placement.^11,13,14^ However, their routine clinical application in lip augmentation and their impact on complication rates and patient-reported outcomes remain inconsistently documented. These gaps underline the need for a systematic evaluation of the available literature that specifically compares different injection approaches for HA lip augmentation.

Therefore, the main objective of this systematic review is to evaluate the safety of HA injection techniques for lip augmentation—specifically, the linear/threading, retrograde/fanning, and microdeposit approaches—used individually or in combination. Secondary objectives include assessing patient satisfaction, injection volume, and exploring potential associations between the device used (needle vs cannula), the brand/type of HA, and the incidence and timing of complications, in order to provide an evidence-based framework for safer and more standardized clinical practice in facial aesthetic medicine.

## METHODS

This study was designed as a systematic review and was conducted in accordance with the PRISMA 2020 statement. The review was conceived, conducted, and reported by a single investigator (Victor Rene Miranda Cerna, MD). The protocol was prospectively registered in PROSPERO on August 16, 2025 (registration ID: CRD420251123328), and no deviations from the protocol were made.

### Eligibility Criteria (Population, Intervention, Comparator, Outcomes)

Population, intervention, comparator, outcomes (PICO) were defined a priori as follows:

- Population (P): Adults (≥18 years) undergoing lip augmentation with HA fillers.
- Interventions (I): Injection using the *linear*/*threading* technique (continuous retrograde deposition along a linear tract; performed with a needle or cannula).
- Comparators (C): Injection using *retrograde*/*fanning* techniques or *microdeposit* (*microdroplet*) techniques, applied individually or in combination.
- Outcomes (O):
  - Primary outcome: Safety, defined as the incidence, type, and timing of procedure-related complications (eg, bruising, prolonged edema, asymmetry, nodules, intravascular events, and infection). When reported, complications were categorized by predefined timing strata as immediate (during the procedure to ≤24 h), early (>24 h to ≤14 days), or late (>14 days). When timing was not explicitly reported, events were coded as not reported (NR).
  - Secondary Outcomes: (1) patient satisfaction with aesthetic results (Global Aesthetic Improvement Scale [GAIS], Visual Analog Scale [VAS], FACE-Q, Likert-type scales, or study-specific instruments); (2) injection volume (total milliliters per lip or per session); (3) association between injection technique and occurrence of complications; and (4) association between HA brand/type and complications.

**Figure 1.**
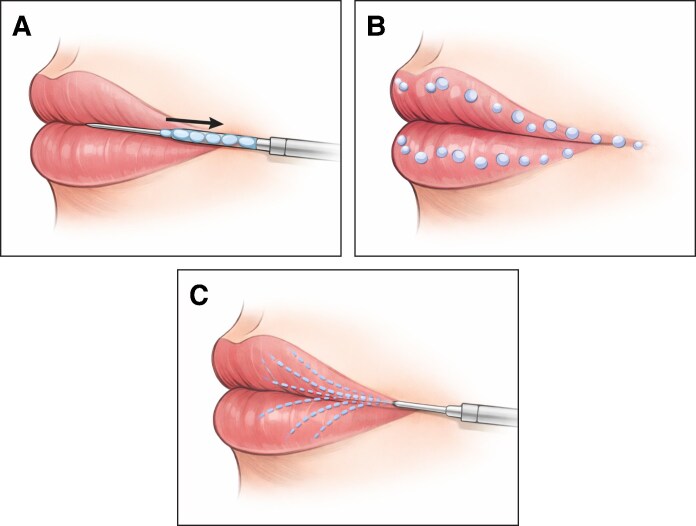
Schematic illustration of hyaluronic acid lip injection patterns. (A) Linear retrograde threading (continuous retrograde deposition along a linear tract). (B) Microdeposit (microbolus) technique (multiple small aliquots placed to refine contour, projection, and texture). (C) Fan/fern pattern (radial distribution from a single-entry point). Patterns may be used individually or combined within the same session depending on the treated subunit and aesthetic objective.

Although the original inclusion prioritized comparative designs with at least 2 injection techniques, a small number of noncomparative studies using a single, clearly described technique were retained when they provided relevant data on safety, injected volume, or patient-reported outcomes for one of the target approaches (linear retrograde, threading, microdeposit technique, and fan/fern patterns). These studies were included in the qualitative synthesis only and did not contribute to any direct comparative analysis between techniques. For conceptual clarity, the main injection patterns considered in this review (linear retrograde threading, microdeposit, and fan/fern patterns) are illustrated in Figure 1.

### Study Designs

Eligible designs included randomized or nonrandomized comparative clinical studies, prospective or retrospective cohort studies, and controlled clinical studies that compared at least 2 injection techniques or provided extractable data for at least one of the target techniques. Noncomparative case series were included only when they reported relevant safety or satisfaction outcomes for a clearly defined injection technique in lip augmentation. Case reports, narrative reviews, expert opinions, conference abstracts without full data, animal studies, and in vitro studies were excluded.

Secondary evidence syntheses (systematic reviews and/or meta-analyses) were eligible only to provide contextual safety and outcome information when technique-specific clinical data were reported and were not pooled with primary clinical studies to avoid duplication of patient-level data. Where a secondary synthesis may have overlapped with included primary studies, findings from the synthesis were used descriptively without double-counting events or sample sizes.

### Information Sources and Search Strategy

A comprehensive literature search was conducted in PubMed/MEDLINE, Embase, Cochrane Library, LILACS, SciELO, and Scopus for studies published from January 1, 2010, to August 16, 2025. The search was limited to studies published in English or Spanish.

The search strategy combined controlled vocabulary (MeSH/Emtree) and free-text terms related to lip augmentation and HA injection techniques. A sample PubMed strategy was:

(“hyaluronic acid” OR hyaluronate OR “HA filler*”) AND (lip OR labial OR perioral) AND (augmentation OR enhancement OR “dermal filler”) AND (linear OR threading OR “retrograde” OR fanning OR “micro-deposit” OR microdeposit* OR microdroplet). The reference list of the included articles and relevant reviews was manually screened to identify additional eligible studies. Bibliographic management and duplicate removal were performed using ZOTERO.

### Study Selection

Records retrieved from all databases were imported into a reference manager, and duplicates were removed using automated and manual procedures. Titles and abstracts were then screened for relevance to lip augmentation with HA and the use of at least 1 target injection technique. At this stage, studies were excluded primarily for the following reasons:

- Non-lip aesthetic procedures
- Use of non-HA fillers
- Noninterventional designs (eg, narrative reviews, expert opinions, and editorials)
- Pediatric populations
- Lack of description of the injection techniques
- Focus on reconstructive or surgical procedures rather than cosmetic augmentation

**Figure 2.**
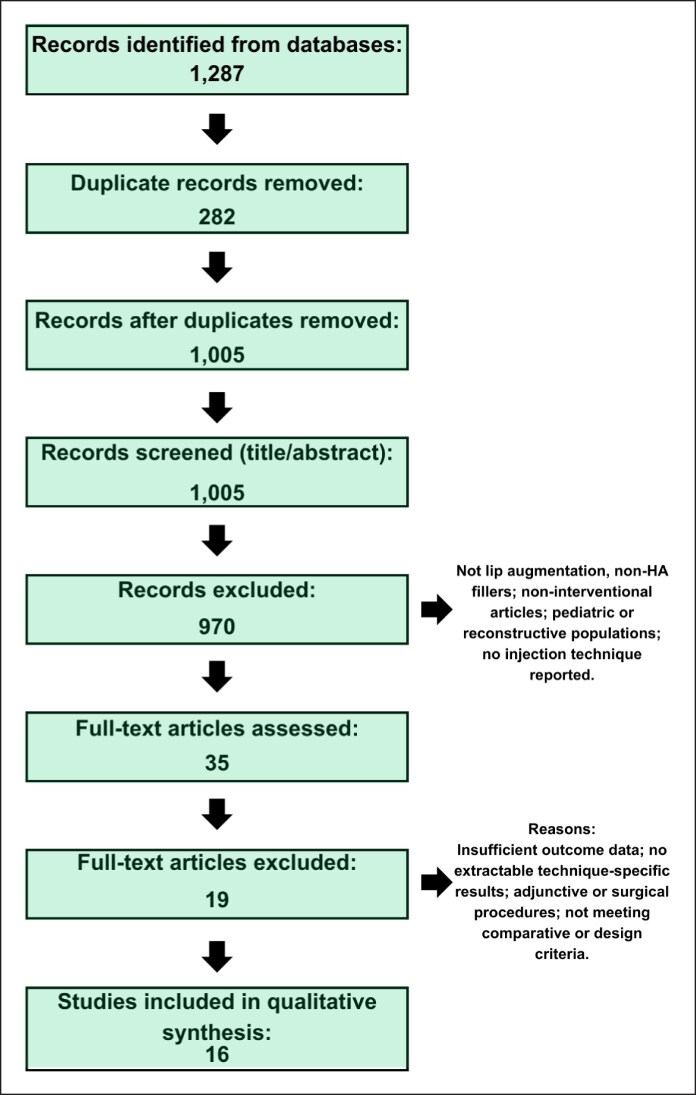
PRISMA 2020 flow diagram of study selection. Records identified through searching (*n* = 1287). After duplicate removal (*n* = 282), 1005 records remained for title/abstract screening; 970 records were excluded. Thirty-five full-text articles were assessed for eligibility and 19 were excluded. Sixteen studies were included in qualitative synthesis.

Full texts were retrieved for potentially eligible reports and assessed against the predefined inclusion and exclusion criteria. Studies were excluded at the full-text stage when outcome data were insufficient, technique-specific results were not extractable, adjunctive/surgical procedures precluded attribution of outcomes to injection technique, or design/comparative criteria were not met. Reasons for exclusion at the full-text stage were recorded and will be presented in a separate table accompanying the PRISMA flow diagram (Figure 2).

### Data Extraction

Data extraction was performed by the same investigator using a standardized form. Because the review was conducted by a single reviewer, data extraction was performed in 2 separate passes at different time points; extracted variables were cross-verified against the original full texts before synthesis and table finalization to minimize transcription and classification errors. The following data were collected:

- Study characteristics: first author, year of publication, country, design, sample size, follow-up.
- Procedure characteristics: injection technique(s) evaluated (linear/threading, retrograde/fanning, microdeposit), device used (needle vs cannula), tissue plane, lip region treated (upper/lower/both), and HA brand/type or rheological profile where reported, including needle–cannula gauge and length when explicitly reported.
- Safety outcomes: type and number of complications, timing of onset (immediate, early, or late when available), and any serious adverse events (eg, vascular compromise, necrosis, and infection). Timing strata were predefined as follows: immediate (during the procedure to ≤24 h), early (>24 h to ≤14 days), and late (>14 days). When timing was not explicitly reported, it was coded as NR.
- Patient-reported outcomes: Satisfaction scales used and reported scores or response distributions.
- Injection volume (mL): Reported volume per lip or per session (mL), expressed as means ± standard deviation or medians with interquartile range when available.

When reporting was unclear or missing, variables were coded as NR rather than inferred. When outcomes were reported only as absolute numbers, complication rates were calculated as *n*/*N* (%).

Level of evidence assessment: Each included primary clinical study was assigned Level of Evidence (I-IV) using the American Society of Plastic Surgeons (ASPS) Evidence Rating Scale for Therapeutic Studies.^15^ Level I includes high-quality randomized controlled trials (RCTs); Level II includes lesser-quality RCTs or prospective comparative/cohort studies; Level III includes retrospective comparative/cohort studies or case–control designs; and Level IV includes case series (pre/post or posttest only). In this review, randomized trials were classified as Level II when methodological limitations (eg, incomplete reporting of allocation concealment and/or blinding) precluded designation as high-quality RCTs. Studies consistent with Level V (expert opinion, case reports/clinical examples, or bench/physiology evidence) were not eligible for inclusion. Secondary evidence syntheses (systematic reviews/meta-analyses) were summarized separately and were not assigned a therapeutic Level of Evidence.

### Risk of Bias Assessment

Risk of bias was assessed by the single investigator according to study design. RCTs were evaluated using the Cochrane RoB 2 tool; nonrandomized and observational comparative studies were assessed with ROBINS-I. For noncomparative case series, a structured checklist adapted from validated tools for case series (eg, clarity of inclusion criteria, consecutive patient recruitment, and completeness of outcome reporting) was applied.

Each domain within the respective tools was rated, and an overall risk-of-bias judgment (low, some concerns, or high risk) was assigned for each study. The impact of risk of bias on the interpretation of findings was considered qualitatively in the synthesis. The fact that assessments were performed by a single reviewer represents an additional limitation and may increase the risk of subjective bias. IRB approval and informed consent were not required because this study is a systematic review of previously published literature and did not involve new human participant recruitment or collection of identifiable patient data.

### Data Synthesis and Statistical Analysis

Given the anticipated methodological and clinical heterogeneity among studies (differences in study design, sample size, injection protocols, filler products, and outcome measures), the primary approach to synthesis was narrative. Primary clinical studies were synthesized as the main evidence base; secondary evidence syntheses were summarized separately and used only for contextual interpretation (eg, rare or delayed events), without contributing to pooled analyses or to denominators used for summary statistics. Where ≥2 studies reported comparable outcomes for the same injection technique or device, findings were summarized descriptively. Formal meta-analysis (eg, pooled proportions or standardized mean differences) was not performed because outcome definitions, timing windows, and satisfaction instruments were heterogeneous and often insufficiently reported for valid pooling.

Planned subgroup analyses included injection technique (linear/threading, retrograde/fanning, microdeposit), device type (needle vs cannula), lip treated (upper vs lower), and HA brand/type where sufficient data were available. Meta-regression was not performed because of the limited number of studies and the small sample sizes. No formal assessment of publication bias (eg, funnel plots) was conducted for the same reason.

## RESULTS

### Study Selection

The database search identified 1287 records. After removal of duplicates (*n* = 282), 1005 records underwent title/abstract screening, and 35 full texts were assessed for eligibility. Nineteen studies were excluded at full-text review, and 16 studies met inclusion criteria and were included in the qualitative synthesis (Figure 2).

### Characteristics of the Included Studies

The 16 included articles collectively reported data on 3692 adult patients undergoing HA injection techniques for lip augmentation. Of these, 14 were primary clinical studies and were classified according to level of evidence using the ASPS therapeutic study framework (Levels I—IV): Level II included 8 randomized or randomized controlled clinical trials; Level III comprised 3 nonrandomized comparative studies; and Level IV consisted of 3 cohort studies. The remaining 2 articles were secondary evidence syntheses (1 systematic review and 1 meta-analysis/systematic review) and were summarized separately for context without assignment of a therapeutic Level of Evidence and without integration into primary-study counts or quantitative summaries.

Across the included studies, the main injection approaches evaluated were linear retrograde techniques, retrograde/fanning patterns, and bolus or microdeposit techniques. Study characteristics, including sample size, HA product, volume injected, follow-up, satisfaction measures, and reported complications, are summarized in Supplemental Table 1.

### Risk of Bias

The overall methodological quality of the included studies was variable. According to the RoB 2 tool, RCTs and randomized clinical trials generally showed some concerns related to the randomization process and lack of blinding of outcome assessors; allocation concealment and reporting of prespecified outcomes were often insufficiently described. Nonrandomized and observational comparative studies, appraised with ROBINS-I, frequently presented moderate to serious risk of bias because of confounding, selection of participants, and lack of adjustment for baseline differences. Cohort studies had additional limitations related to the absence of control groups, nonconsecutive recruitment, and incomplete reporting of outcomes.

These 2 evidence syntheses (systematic review and meta-analysis) also reported methodological limitations and noted limitations in the underlying evidence base, mainly because of observational designs, small sample sizes, and potential publication bias. Taken together, these sources of bias, along with heterogeneity in techniques, products, and outcome measures, limit the interpretability of the finding of this review and should be taken into account when interpreting the results. The detailed risk-of-bias assessment for each study is summarized in Table 1.

**Table 1.** Risk of Bias Assessment of Included Studies

Study	Study design	Tool used	Overall risk-of-bias	Main concerns
Keramidas et al^1^	Clinical trial (nonrandomized)	ROBINS-I	Moderate	Confounding, selection of participants
Rho et al^2^	Randomized single-blinded study	RoB 2	Some concerns	Allocation concealment, blinding, reporting of outcomes
Hilton et al^3^	Randomized clinical trial	RoB 2	Some concerns	Randomization, deviations from intended interventions
Gonzalez et al^4^	Cohort study	ROBINS-I	Serious	Serious confounding, lack of control group, incomplete adjustment for baseline differences
Czumbel et al^5^	Meta-analysis and systematic review	N/A	N/A	Not formally assessed in this table
Kim^6^	Cohort study	ROBINS-I	Serious	Serious confounding, nonconsecutive inclusion, incomplete outcome reporting
Müller et al^7^	Randomized controlled trial	RoB 2	Some concerns	Randomization, blinding, missing outcome data
Nikolis et al^8^	Prospective clinical trial	ROBINS-I	Moderate	Confounding, blinding, outcome assessment
Beer et al^9^	Randomized clinical trial	RoB 2	Some concerns	Randomization, blinding, outcome assessment
Raspaldo et al^10^	Randomized controlled trial	RoB 2	Some concerns	Randomization process, incomplete reporting
Smith et al^11^	Randomized clinical trial	RoB 2	Some concerns	Randomization, blinding, outcome assessment
Weiss et al^12^	Randomized, controlled, evaluator-blinded, multicenter study	RoB 2	Some concerns	Randomization process: incomplete reporting of allocation concealment and blinding procedures
Bertossi et al^13^	Randomized clinical trial	RoB 2	Some concerns	Randomization, blinding, outcome assessment
Coppini et al^14^	Systematic review	N/A	N/A	Not formally assessed in this table
Buhsem^16^	Randomized controlled trial	RoB 2	Some concerns	Randomization process, blinding of outcome assessment
Hilton et al^17^	Controlled clinical trial (nonrandomized)	ROBINS-I	Moderate	Selection bias, outcome measurement

RCT, randomized controlled trial; RoB 2, Cochrane risk-of-bias 2 tool; ROBINS-I, risk-of-bias in nonrandomized studies of interventions; N/A, not applicable. Overall risk-of-bias judgments (low, some concerns, or high for RoB 2; low, moderate, serious, or critical for ROBINS-I) were assigned according to the guidance of each tool. This assessment should be interpreted in the context of the narrative description provided in the Results section.

### Injection Instruments and Techniques

Information on the injection instrument (needle vs cannula) was explicitly reported in all 14 primary clinical studies. Of these, 9 studies (64.3%) used needle only, whereas 5 studies (35.7%) used a combination of needle and cannula; no primary study used cannula alone as the exclusive device. The 2 secondary evidence syntheses did not clearly specify the injection instrument and were summarized separately. Needle and cannula specifications (gauge and length) were inconsistently reported; when available, these details are summarized in Supplemental Table 1 and otherwise coded as NR. Where reported, gauge and length were extracted and presented alongside the corresponding injection pattern (eg, linear retrograde/threading, fan/fern, or microdeposit) to facilitate technique-level interpretation.

With respect to injection technique, linear retrograde injection (including linear threading/retrograde technique) was the most frequently reported approach, described in 12 studies (75%). Bolus or microdeposit techniques were reported in 2 studies (12.5%), and serial puncture, cross-hatching, or fan/fern patterns were described in 4 studies (25%). In several studies, >1 technique was used within the same protocol; therefore, percentages for injection techniques are overlapping and do not sum to 100%.

### Injected Volume

Information on injected volume was available in all 14 primary clinical studies. When studies reported per-lip volumes, these summed to derive per-session totals for comparability; studies reporting only nonextractable total treatment volume were retained descriptively but were not used to calculate the mean. The mean injected volume was ∼1.57 mL per treatment session, with a range from 0.6 to 3.0 mL. Most studies reported total volumes between 1 and 2 mL per session, which generally corresponded to moderate lip augmentation. Higher volumes (up to 3 mL) were used when both lips were fully treated or when multiple techniques were combined. Some studies reported separate volumes for each lip, whereas others provided only the total volume per session; when per-lip volumes were reported, values were summed to derive a session total for comparability, which may contribute to minor variability in the calculated average.

### Safety Outcomes

All 14 primary clinical studies reported safety data, although the level of detail varied. The 2 secondary evidence syntheses also reported safety outcomes and were summarized separately as contextual evidence. A qualitative summary of safety and patient satisfaction outcomes according to injection technique and instrument is provided in Table 2. Overall, adverse events were predominantly mild and transient, consisting mainly of edema, ecchymosis/hematoma, local pain, and transient nodularity or induration at the injection site.

**Table 2.** Qualitative Summary of Safety and Satisfaction According to Injection Technique and Instrument in Hyaluronic Acid Lip Augmentation

Technique/instrument	Typical volume per session	Safety profile (qualitative)	Patient satisfaction (qualitative)	Notes
Linear retrograde threading (needle)	∼1-2 mL (up to 3 mL in some protocols)	Mild, transient edema and bruising are common; occasional nodularity or hematoma; no ischemic or vision-threatening events reported in the included clinical series	Generally high; most trials report ≥80%-90% of patients rated as “improved” or “much improved”	Most frequently used pattern and backbone of many protocols
Linear retrograde ± serial puncture/fan/fern (needle)	∼1-2 mL	Similar to linear retrograde alone; transient edema and ecchymosis predominate; occasional palpable irregularities with more focal boluses	High; GAIS, FACE-Q, or Likert score usually in the upper range	Serial puncture and fan/fern are mainly used for contour refinement and specific lip-perioral subunits
Linear retrograde + bolus/microdeposit (needle ± cannula)	∼1.5-2.5 mL (often including touch-up or combined lip-perioral treatment)	Frequent short-term swelling and bruising; no necrosis or vascular occlusion reported in mixed needle–cannula protocols; rare delayed inflammatory reactions described in broader needle-based series	High; FACE-Q and Likert score close to the upper end of the scale, with ≈74%-100% of patients reporting “high” or “great” satisfaction	Microdeposits and small boluses are useful for local projection and contour refinement
Needle-only vs needle plus cannula (all techniques)	Overlapping ranges; most protocols use ∼1-2 mL regardless of instrument	No consistent difference in overall adverse-event patterns; both strategies associated with predominantly mild, self-limited events when used by experienced injector; serious ischemic events not documented in the included clinical series	No clear difference; high-satisfaction rates reported in both groups	Instrument choice alone does not appear to determine safety or satisfaction; technique and injector expertise are likely more relevant

FACE-Q, FACE-Q patient-reported outcome measure; GAIS, Global Aesthetic improvement Scale; HA, hyaluronic acid. This table provides a qualitative synthesis based on the 16 included studies and does not represent a quantitative meta-analysis.

Adverse events were additionally categorized by predefined timing strata: immediate (≤24 h), early (>24 h to ≤14 days), and late (>14 days), when explicitly reported. However, the timing of onset was inconsistently documented across studies, and several reports did not provide sufficient detail to classify events by time; these cases were coded as NR. A summary of adverse events by timing stratum is provided in Table 3.

**Table 3.** Adverse Events by Predefined Timing Strata (Immediate, Early, and Late), as Reported in Included Studies

Study	Follow-up/assessment schedule (as reported)	Immediate (≤24 h) *n*/*N* (%) + event types	Early (>24 h to ≤14 days) *n*/*N* (%) + event types	Late (>14 days) *n*/*N* (%) + event types	Timing NR (yes/no)	Notes (strictly as reported)
Keramidas et al^1^	NR	NR	NR	NR	Yes	Complications described, but timing not explicitly stated in extractable form
Rho et al^2^	NR	NR	*N* = 3 events described: 2 bruises (Group B) + 1 local swelling (Group A), resolved within a week without intervention	NR	Yes (partial)	Timing given only as “resolved within a week,” no further stratified counts/time points
Hilton et al^3^	14 days diary + longer follow-up	NR (diary used for early reactions)	Most local reactions resolved within 14 days (counts by timing not provided)	1 patient: redness after Day 14, continued 32 days	Yes	Paper states early local reactions tracked by diary; “ongoing after Day 14” considered AEs. Only the redness for 32 days is clearly classifiable as late with a discrete duration.
Gonzalez et al^4^	NR	—	—	—	No	No complications reported (timing not applicable).
Czumbel et al^5^	Includes studies with follow-up up to months	NR	NR	NR	Yes	Mentions AEs resolving in “few weeks” in general terms; not consistently stratified per study/technique for immediate/early/late counts
Kim^6^	NR	—	—	—	No	No major complications reported (timing not applicable)
Müller et al^7^	NR	NR	NR	NR	Yes	AEs described as mild/temporary, but no explicit days/weeks captured for timing classification
Nikolis et al^8^	NR	NR	NR	NR	Yes	Timing not extractable
Beer et al^9^	Multi-visit follow-up (includes early monitoring)	TEAEs described as beginning within a day; severe TEAEs: lip swelling 2% (as stated)	NR	NR	Yes	Onset is immediate (≤24 h) per text; duration by days not clearly extractable
Raspaldo et al^10^	Visits at Day 1 and Day 14 after each treatment; 1 and 3 months after last treatment; ISRs recorded in a 30-day subject diary	NR	Most ISRs lasted ≤14 days (as reported)	ISRs lasting 15-30 days: 23.4% (Volbella) and 25.6% (Restylane-L); most commonly lumps/bumps and firmness; swelling lasting 15-30 days: 0.8% vs 4.5%	No	Timing reported as ISR duration categories (≤14 days; 15-30 days)
Smith et al^11^	Follow-up includes longer-term assessment (months)	NR	NR	NR	Yes	Timing not reported
Weiss et al^12^	NR	NR	Lumps, hematoma (duration <7 days, as reported)	NR	Yes (partial)	Duration reported; onset not specified
Bertossi et al^13^	NR	NR	NR	NR	Yes	Timing not reported
Coppini et al^14^	Long-term (case-based ADR timing reported)	NR	NR	Delayed lip ADRs/granulomatous reactions: median 24 months (range reported in paper)	No	This is a review on ADRs, timing is explicitly long-term (months/years)
Buhsem^16^	NR	—	—	—	No	No complications reported (timing not applicable)
Hilton et al^17^	Early AEs assessed to Day 14; post-day 14 AEs assessed up to 24 weeks	NR	Early-onset AEs reported up to Day 14 (counts by stratum not provided in extracted text)	Post-Day 14 AEs assessed up to 24 weeks (counts by stratum not provided in extracted text)	Yes	Timing framework (Day 14 vs post-Day 14) is explicit; counts by timing stratum are not clearly extractable from provided text alone

Immediate refers to events occurring during the procedure or within ≤24 h; early refers to events occurring >24 h to ≤14 days; and late refers to events occurring >14 days. NR indicates that the timing of onset was not reported or was insufficiently detailed to classify the event into a predefined stratum. Event frequencies are presented as reported (*n*/*N*, %), and percentages were calculated only when absolute numbers were available. ADR, adverse drug reactions; AE, adverse events; TEAEs, treatment-emergent adverse events.

No included study explicitly reported standardized management protocols for serious complications (eg, vascular compromise or foreign-body granulomas). Accordingly, management strategies for vascular compromise or suspected foreign-body granulomatous reactions could not be extracted or compared across techniques and were coded as NR.

Information on the injection instrument was explicitly available in all 14 primary clinical studies. Among these, 9 studies using needle only and 5 studies using a combination of needle and cannula showed broadly similar patterns of early, self-limited adverse events. In the needle + cannula studies, complications were mostly limited to short-term edema and bruising; for example, 2 cohort/controlled studies using linear retrograde and bolus techniques with both devices reported high rates of transient edema (up to 88%) and hematoma (around 17%), but no major or long-lasting complications. A small cohort directly comparing needle and cannula in linear retrograde injection described no complications in either group.

In the primary clinical studies, late or more serious adverse events were uncommon but reported, most often in needle-only protocols or in studies with heterogeneous or incompletely specified techniques. For example, 1 randomized clinical trial using needle only for linear retrograde/anterograde threading and serial puncture reported severe treatment-related adverse events (marked lip swelling) in 2% of patients. Secondary evidence syntheses (systematic review/meta-analysis) also described late and/or less common adverse events (including granulomatous reaction and herpes labialis); however, these findings were summarized as contextual evidence and were not integrated with primary-study results to avoid double counting.

When outcomes were examined by injection techniques, mild adverse events were observed across all approaches—linear retrograde, anterograde/retrograde threading, serial puncture, fan/fern patterns, and bolus/microdeposit techniques. Linear retrograde injection with needle was the most commonly studied technique and was associated mainly with edema, bruising, and occasional pain or nodularity; severe hematomas and marked inflammation were reported in at least 1 large clinical series using this approach. Studies combining linear retrograde and bolus/microdeposit techniques, frequently delivered with needle plus cannula, tended to show high rates of short-term swelling but did not report necrosis, vascular occlusion, or vision-threatening ischemic events. Nevertheless, adverse-event definitions and reporting windows varied, and absence of reporting should not be interpreted as absence of occurrence.

Taken together, the available evidence indicates that the most commonly reported adverse events after HA lip augmentation—across instruments and techniques—were mild and self-limited. However, inconsistent reporting and limited follow-up in several studies mean that underreporting of rare or delayed complications cannot be excluded. The occurrence of granulomatous reactions and severe edema in some needle-based series, the heterogeneity of products and protocols, and the lack of head-to-head trials specifically powered to compare safety across instruments and techniques prevent definitive conclusions about the superiority of any single approach.

### Patient Satisfaction

Most primary clinical studies assessed patient-reported outcomes using GAIS, FACE-Q modules, VASs, or study-specific Likert-type ratings. Despite heterogeneity in instruments and follow-up intervals, overall satisfaction was consistently high across the primary clinical studies. A structured mapping of satisfaction instruments, validation status, and assessment time points is provided in Table 4. Among needle-only protocols, GAIS-based ratings and study-specific satisfaction scales generally indicated improvement or high satisfaction at short- to mid-term follow-up, whereas FACE-Q-based studies reported high satisfaction at follow-up time points. Variability in reported scores likely reflects differences in baseline anatomy, patient expectations, product selection, and the fact that many protocols combined multiple techniques within the same session. Direct cross-study comparisons of absolute satisfaction scores were not appropriate because of nonequivalent scales and variable reporting.

**Table 4.** Mapping of Patient Satisfaction Outcome Measures across Included Studies (Instrument Type, Validation Status, and Assessment Time Points)

Study	Satisfaction instrument (as reported)	Validated	Rater	Time points (as reported)	Notes
Keramidas et al^1^	Written patient questionnaire + GAIS (5-point)	GAIS: NR/not specified; questionnaire: NR/study specific	Patient (questionnaire/GAIS marking)	Immediately after the procedure; 15 days later	From 833 patients, all completed at least 1 follow-up questionnaire; GAIS distribution reported using the study's wording (eg, exceptional): 92.4% “exceptional,” 7.56% “very improved/improved,” 0% “no result/worse.”
Rho et al^2^	Patient satisfaction questionnaire (ad hoc; 0-3; domains: volume shape, attractiveness, naturalness); GAIS (−1 to 3); LFS (0-4)	Patient satisfaction: NR/ad hoc; GAIS/LFS: standardized scales	Patient (satisfaction); blinded evaluators (LFS/GAIS; photograph based)	4 and 12 weeks	Patient overall satisfaction means: 2.8 vs 2.7 (Week 4) and 2.6 vs 2.6 (Week 12); naturalness: 2.9 vs 2.6 at Week 4 (Group A higher)
Hilton et al^3^	Patient satisfaction questionnaire (items on satisfaction with lip fullness, attractiveness, natural look, facial balance; plus willingness to repeat/recommend)	NR as validated (study-specific questionnaire)	Patient reported	Month 1 and Month 12 (questionnaire responses); recovery acceptability at 2 weeks also reported	Month 1: 96% somewhat/very satisfied with lip fullness; 86% somewhat/fully agreed they felt more attractive; 96% somewhat/fully agreed lips had a natural look; 86% somewhat/fully agreed treatment added balance to facial features. Month 12: would repeat treatment (HA-RK 96% vs HA-JV 91%); would recommend to a friend (HA-RK 100% vs HA-JV 95%); felt more attractive (HA-RK 85.7 vs HA-JV 86.2%)
Gonzalez et al^4^	Custom subjective satisfaction scale (1-5)	No (study-specific)	Patient	NR (reported as postprocedure; exact assessment time point not specified)	Follow-up period reported as 8 months; satisfaction not stratified by device/technique (cannula vs needle)
Czumbel et al^5^	NR (NR as a patient satisfaction instrument)	N/A	N/A	N/A	Meta-analysis/systematic review assessing effectiveness (responder rate: ≥1-point lip fullness increase) and adverse events; satisfaction instruments were not mapped/reported as a standardized patient satisfaction outcome in this paper
Kim^6^	GAIS (patient rated; −1 to +3).	NR/not specified	Patient	1 week; 1 month; 3 months	Mean GAIS: 2.8 at 1 week (reported as 90% “extreme satisfaction”), 2.4 at 1 month, and 1.9 at 3 months
Müller et al^7^	FACE-Q (satisfaction with lips); GAIS (investigator and patient); additional patient satisfaction questionnaire (“pleased with the result”)	FACE-Q: yes; GAIS: standardized scale; satisfaction questionnaire: NR/study specific	Patient (FACE-Q, GAIS, questionnaire); investigator (GAIS)	Week 6; follow-up at Months 6, 12, and 18	Pleased with the result (somewhat + definitely agree): 99.1% at Week 6, 96% at Month 6, 69.1% at Month 18. FACE-Q satisfaction with lips mean converted score 90.9/100 at Week 6; “lips suited their face” (somewhat + definitely satisfied): 92.7% at Month 6 and 67.3% at Month 18
Nikolis et al^8^	NR (no patient satisfaction instrument described)	NR	NR (objective imaging endpoints reported)	Baseline to Week 8 (assessment at Week 8; injection included initial + optional touch-up)	Satisfaction outcomes not extractable; NR for satisfaction-specific instrument
Beer et al^9^	GAIS (7-point: 3 to −3)	Standardized scale; NR as a validated PROM	Patient + treating investigator	Weeks 2, 4, 8, 12, 16, 20, and 24	GAIS distribution summarized as “improved or better” (upper + lower lips): Week 8: 90.1% (patient) and 96.5% (investigator); Week 24: 75.6% (patient) and 83.8% (investigator); GAIS categories include very much improved (3) to very much worse (−3)
Raspaldo et al^10^	FACE-Q recovery early life impact; FACE-Q recovery early symptoms; FACE-Q satisfaction with lips; FACE-Q satisfaction with outcome; investigator overall satisfaction (0-10 scale)	Yes (FACE-Q); investigator 0-10 scale: NR/unclear	Patient (FACE-Q); investigator (0-10)	FACE-Q recovery modules: Days 1 and 14 after each treatment; FACE-Q satisfaction modules: through Month 3	Patients were significantly more satisfied at Month 3 on FACE-Q satisfaction with lips and satisfaction with outcome (between-group comparisons reported)
Smith et al^11^	GAIS	NR/not specified in this report	NR (context suggests patient/evaluator GAIS, but not explicitly detailed here)	NR (not explicitly reported for GAIS in this Supplemental Material)	Study focuses on functional lip safety assessments; for risk-benefit context, 99% of patients with any abnormal lip assessments had GAIS rating of “improved or better,” and 77% elected retreatment
Weiss et al^12^	FACE-Q (satisfaction with lips + appraisal of lip lines; Rasch-transformed total score)	Yes (FACE-Q)	Patients (patient reported)	Baseline; follow-up visits included Week 8 (primary endpoint timing) and continued through Week 48	Study also reports “aesthetic improvement” and “patient satisfaction” as secondary endpoints; optional touch-up at Week 4
Bertossi et al^13^	Patient self-assessment using a 7-point Likert scale (0-6)	NR/study specific (NR as validated)	Patient	4 weeks	Reported by age group: 20-34 years, mean 4.9; 35-45 years, means 4.8; ≥46 years, mean 4.8; 92% satisfied (score 4-6). Study follow-up duration: 6 months (as reported), but satisfaction was measured at 4 weeks
Coppini et al^14^	NR (not assessed)	N/A	N/A	N/A	Systematic review focused on ADRs after lip augmentation with dermal fillers; patient satisfaction outcomes/instruments were NR
Buhsem^16^	Custom patient satisfaction questionnaire (0-5)	NR/unclear	Patient	3 weeks (last follow-up day)	Four groups; mean satisfaction (0-5): G1 4.78, G2 3.70, G3 4.15, G4 3.85 (as reported)
Hilton et al^17^	Patient satisfaction questionnaires; GAIS (patient-blinded and blinded evaluator assessed)	Satisfaction questionnaire: NR/study specific; GAIS: standardized scale	Patient (questionnaires + GAIS); blinded evaluator (GAIS, photograph based)	Satisfaction questionnaires: screening/baseline, Days 0 and 14, and Weeks 4, 12, 24; GAIS: Day 14, Weeks 4, 12, and 24	Patient satisfaction with lip appearance increased from baselines (20% somewhat/very satisfied) to Day 14 (100% vs 90%) and Week 12 (95% vs 90%), with 79% vs 70% still somewhat/very satisfied at Week 24; additional questionnaire items included attractiveness, “natural look,” and willingness to repeat/recommend at Week 24

ADR, adverse drug reactions; FACE-Q, Facial Clinometric Evolution Questionnaire; GAIS, Global Aesthetic Improvement Scale; LFS, Lip Fullness Scale; NR, not reported; N/A, not applicable. “Validated instrument” indicates whether the study explicitly used a validated patient-reported outcome measure (eg, FACE-Q). study-specific questionnaires and unvalidated scales were classified as NR/unclear validation was reported. Time points reflect when satisfaction outcomes were assessed as reported in each study.

Studies in which needle and cannula were used in combination, often within mixed-technique protocols, also reported favorable patient satisfaction across the primary clinical studies. Across these studies, satisfaction was generally reported as high using study-specific Likert-type ratings and/or GAIS-based assessments, although scoring anchors and reporting formats varied. A randomized trial comparing different HA products injected with needle and cannula reported high FACE-Q satisfaction scores across groups. No study reported a clear reduction in satisfaction associated specifically with the use of cannula.

When satisfaction was analyzed by injection pattern, linear retrograde techniques (with or without adjunctive serial puncture or fan/fern patterns) again predominated and were associated with high patient-reported outcomes in most series. Studies incorporating bolus/microdeposit techniques—usually combined with linear retrograde passes and sometimes delivered through cannula—also reported high patient satisfaction using FACE-Q modules or study-specific measures.

Because satisfaction measures were not standardized, follow-up periods varied, and many studies combined >1 injection pattern and device within the same protocol, formal comparative analyses by instrument or technique were not feasible. Nevertheless, the narrative synthesis suggests that patient satisfaction after HA lip augmentation is generally high across reported instruments (needle alone or needle plus cannula) and injection patterns; however, heterogeneity in measures, follow-up, and mixed-technique protocols limits technique- or device-specific inferences.

## DISCUSSION

This systematic review synthesized the available evidence on HA injection techniques for lip augmentation, with a primary focus on safety and secondary outcomes including patient satisfaction, injected volume, and potential associations with injection device and technique. Across 16 included articles involving 3692 adult patients, HA lip augmentation was consistently associated with clinically meaningful aesthetic improvements, high levels of patient-reported satisfaction, and a predominantly mild and transient adverse-event profile.^1-14,16,17^ However, the underlying evidence is heterogeneous and methodologically limited, which constrains the strength of any comparative statements regarding specific instruments or techniques.^1-6,8-11^

The present findings are consistent with the broader literature on HA fillers in facial harmonization, where sustained improvements in contour and volume are commonly reported for up to 6 to 12 months when appropriate products and conservative volumes are used.^2,3,5,6,9,11,12,17^ In the included studies, the average injected volume for lip augmentation was ∼1.57 mL per session, with most protocols using between 1 and 2 mL.^2,4-6,8,9,11-13^ This range generally produced moderate but clearly visible augmentation while maintaining a favorable safety profile. Higher volumes, up to 3.0 mL, were reserved for more extensive treatments or combined techniques, underscoring the importance of tailoring volume to baseline anatomy and desired outcomes rather than pursuing maximal correction in a single session.^2,5,6,11^

Regarding the primary outcome of safety, adverse events were overwhelmingly mild or moderate, including edema, ecchymosis, and transient nodularity or induration at the injection site.^2,4-6,8-13^ This pattern aligns with what is commonly expected after minimally invasive soft-tissue augmentation. Importantly, among the primary clinical studies included in this review, no cases of vascular occlusion, tissue necrosis, or vision-threatening ischemic events were reported. However, this finding should be interpreted cautiously given heterogeneous reporting standards, variable follow-up, and the likelihood of underreporting of rare but severe outcomes. Isolated granulomatous reactions and marked swelling were described in secondary analyses and evidence syntheses, particularly in needle-based protocols with longer follow-up or less standardized techniques, underscoring that delayed and rare complications remain possible even when immediate outcomes are favorable.^1,3^

With respect to complication prevention and management, because standardized management protocols for serious adverse events were not described in most included studies, complication management could not be synthesized as an outcome. Although serious ischemic events were NR in the included primary clinical studies, lip augmentation with HA carries a recognized risk of vascular compromise and delayed inflammatory reactions. Preventive measures commonly emphasized in the broader HA filler safety literature typically include detailed knowledge of perioral vascular anatomy, conservative injection volumes, slow low-pressure delivery with continuous patient monitoring, and avoidance of high-risk bolus placement. When vascular compromise is suspected, immediate cessation of injection and prompt implementation of established management protocols are recommended, including rapid clinical assessment and prompt administration of hyaluronidase (often using high-dose, pulsed protocols) when indicated, with urgent referral if ocular symptoms are present. For delayed nodules or suspected foreign-body granulomatous reactions, management is typically individualized based on severity and may include observation, anti-inflammatory therapy, hyaluronidase when appropriate, and specialist referral for persistent or complex cases. Future primary studies should report standardized adverse-event management algorithms to enable meaningful comparisons across techniques.

Analysis by injection device suggested that both needle-only and needle plus cannula approaches can be used safely when performed by experienced injectors. Among the 14 primary clinical studies that clearly reported the instrument, needle-only protocols were more common, whereas 5 studies combined needle and cannula within the same treatment strategy.^2,4-14,16,17^ In these mixed-instrument protocols—particularly where cannulas were used for linear retrograde passes or for distributing small boluses or microdeposits—early events such as swelling and bruising were frequent but self-limited, and no major complications were reported.^4,11,12,14^ Conversely, the series and review that described granulomatous reactions or severe edema over longer follow-up involved primarily needle-based techniques and heterogeneous injection patterns.^1-3^ Overall, the current evidence does not demonstrate a clear safety advantage of 1 instrument over another; instead, injector expertise, adherence to anatomical safety guidelines, the use of ultrasound in selected cases, and conservative injection strategies are likely more critical determinants of risk than the isolated choice of needle vs cannula.^2,11,13,14^

The evaluation of injection techniques similarly revealed a heterogeneous landscape. Linear retrograde threading was the most frequently described pattern and served as the backbone of many protocols, often combined with serial puncture, fan/fern passes, or small boluses.^2,4-13^ Across studies, linear retrograde injection provided reproducible improvements in lip volume and contour, with a safety profile dominated by mild edema and bruising.^2,3,6,8-11^ Serial puncture and fan/fern techniques were used to refine shape and address specific subunits, again with mostly transient adverse events.^5,8,9,11^ Bolus injections, particularly when larger or placed in fewer points, were effective for local enhancement but were occasionally associated with more palpable irregularities and transient inflammatory reactions in some series.^2,6,11,12^

Microdeposit techniques were described less frequently but were conceptually appealing for their potential to allow finer control of 3-dimensional contour and texture.^4,6,7,11-13^ In protocols where microdeposits were combined with linear retrograde passes, patient satisfaction was high and adverse events remained predominantly mild.^4-6,11,12^ However, these apparent advantages must be interpreted cautiously: microdeposit-dominant protocols were not consistently compared head-to-head with other techniques, and many studies combined multiple patterns within the same session, making it difficult to isolate the effect of any single approach.^2,4-6,8,9,11-13^ Overall, the available data suggest that several techniques—linear retrograde, serial puncture/fan, bolus, and microdeposit—can all produce favorable outcomes when appropriately selected and executed, but no single technique emerges as definitively superior based on current evidence.

Patient satisfaction, a key secondary outcome, was uniformly high across instruments and techniques. Studies using GAIS, FACE-Q, VASs, and study-specific Likert-type ratings consistently reported aesthetic improvement and high satisfaction at short- to mid-term follow-up.^2,4-6,8-13^ Notably, no consistent reduction in satisfaction was observed in studies that incorporated cannulas in addition to needles, nor in those employing mixed techniques that combined linear, bolus, and microdeposit patterns.^4,5,11,12,14^ Variability in satisfaction scores across individual studies likely reflects differences in baseline anatomy, patient expectations, cultural aesthetic preferences, and details of the injection protocol rather than the impact of a single technical variable.^2,4-6,11-13^

An additional dimension highlighted by this review is the interaction between technique, product characteristics, and patient factors. HA formulations differ in rheology, cross-linking, and cohesivity, and these properties influence tissue integrations, palpability, and durability.^2-6,9,11,12,17^ Likewise, baseline lip structure, perioral soft-tissue quality, age, and Fitzpatrick skin type may modulate both the aesthetic effect and the risk of complications.^2,6,11-13^ Most of the included studies did not stratify outcomes by these factors, limiting the ability to draw nuanced, patient-level conclusions. Nonetheless, the consistency of high satisfaction and acceptable safety across diverse populations supports the principle that HA lip augmentation, when individualized and performed conservatively, is a robust and adaptable modality in facial harmonization.^2,3,5,6,8-13^

From a methodological standpoint, the risk of bias of the included studies was generally moderate to high. Randomized clinical trials often lacked detailed reporting of randomization, allocation concealment, and blinding of outcome assessors.^2,5,6,8-11,17^ Nonrandomized and observational studies were susceptible to confounding, selection bias, and incomplete outcome reporting.^4,8,12-14,16^ Cohort studies frequently lacked control groups and did not systematically use validated, standardized outcome measures.^4,12,13^ Additionally, outcome assessment relied heavily on subjective scales, with limited use of objective tools such as 3-dimensional imaging or ultrasound, except in a few studies.^4,11,13,14^ These limitations reduce the certainty of the evidence and underscore the need for cautious interpretation of technique- and device-specific claims.^1,3^

This review itself has limitations. Study selection, data extraction, and risk-of-bias assessment were conducted by a single reviewer, which introduces a potential risk of selection and extraction bias. Although this risk was mitigated through standardized extraction, 2-pass verification at different time points, and full-text cross-checking of key variables before table finalization (with unclear items coded as NR rather than inferred), residual bias cannot be excluded. The search was restricted to articles in English and Spanish, and publication bias cannot be ruled out. In addition, rare but serious adverse events may be underreported in the included studies, and the absence of reported ischemic complications should not be interpreted as a definitive absence of risk. Finally, substantial clinical and methodological heterogeneity (products, planes, volumes, techniques, follow-up intervals, and outcome instruments) precluded robust quantitative synthesis for most outcomes, necessitating a primarily narrative synthesis. A small number of noncomparative studies using a single technique were retained because they provided relevant safety and outcome data for the target approaches; however, their inclusion may have increased heterogeneity and further limited direct comparisons between techniques.^2,4,12,13^ In addition, inclusion of secondary evidence syntheses introduces a risk of overlapping evidence; to minimize duplication, secondary syntheses were treated as contextual sources and were not combined quantitatively with primary studies or used to recount events.

### Future Directions

Future research should focus on well designed, adequately powered RCTs that directly compare clearly defined injection techniques (eg, linear retrograde-dominant vs microdeposit-dominant protocols) and instrument strategies (needle alone vs needle plus cannula), using standardized safety and satisfaction outcomes.^2,4-6,9,11-13^ The development and adoption of a core outcome set for aesthetic lip augmentation—incorporating validated patient-reported outcome measures (eg, FACE-Q), objective volumetric assessment (eg, 3-dimensional imaging), and standardized safety endpoints—would greatly improve comparability across studies.^3,4,11^ Large prospective registries and multicenter collaborations are also needed to better characterize the incidence and risk factors of rare but serious adverse events, including vascular compromise, delayed inflammatory reactions, and granulomatous responses.^1-3^ In addition, studies integrating imaging tools such as high-resolution ultrasound for preprocedural planning and real-time guidance could help clarify whether these modalities translate into measurable improvements in safety and aesthetic outcomes in routine clinical practice.^11,13,14^

## CONCLUSIONS

Within the limits of the available evidence and acknowledging heterogeneous reporting and follow-up, this systematic review supports that HA lip augmentation is an effective and generally safe procedure, associated with high patient satisfaction and predominantly mild, transient adverse events when performed by experienced injectors. Across studies, available data were insufficient to determine consistent, technique-independent differences between HA products, as product selection, injection protocols, and outcome reporting varied substantially.

Current evidence suggests that a range of injection techniques—including linear retrograde threading, serial puncture and fan patterns, and deposit or microdeposit approaches—can produce favorable aesthetic outcomes with acceptable tolerability. Likewise, both needle-only protocols and strategies combining needle and cannula have demonstrated generally similar short-term safety profiles in the published literature. However, heterogeneity in study designs, products, and outcome measures, together with limited head-to-head comparisons and the potential underreporting of rare or delayed complications, precludes identifying a single superior technique, instrument, or product.

In clinical practice, technique and instrument selection should be individualized based on patient anatomy, aesthetic goals, filler rheology, and injector expertise, while emphasizing conservative volumes and meticulous awareness of perioral vascular anatomy. Further high-quality, standardized, comparative research is needed to clarify technique- and device-specific differences and to support consensus-based best-practice guidelines for HA lip augmentation.

## Supplemental Material

This article contains supplemental material located online at https://doi.org/10.1093/asjof/ojag059.

## Supplementary Material

ojag059_Supplementary_Data

## References

[1] Keramidas E, Rodopoulou S, Gavala MI. A safe and effective lip augmentation method: the step-by-step Φ (phi) technique. Plast Reconstr Surg Glob Open. 2021;9:e3332. doi: 10.1097/GOX.0000000000003332 33680634 PMC7928943

[2] Rho NK, Goo BL, Youn SJ, Won CH, Han KH. Lip lifting efficacy of hyaluronic acid filler injections: a quantitative assessment using 3-dimensional photography. J Clin Med. 2022;11:4554. doi: 10.3390/jcm11154554 35956168 PMC9369503

[3] Hilton S, Sattler G, Berg AK, Samuelson U, Wong C. Randomized, evaluator-blinded study comparing safety and effect of two hyaluronic acid gels for lips enhancement. Dermatol Surg. 2018;44:261–269. doi: 10.1097/DSS.0000000000001282 29059146 PMC5821480

[4] Gonzalez C, Callejas E, Nuñez C, et al Lip volumization with hyaluronic acid: comparative ultrasonographic evaluation of cannula and needle techniques in a multicenter study. Cureus. 2025;17:e79325. doi: 10.7759/cureus.79325 40125164 PMC11928312

[5] Czumbel LM, Farkasdi S, Gede N, et al Hyaluronic acid is an effective dermal filler for lip augmentation: a meta-analysis. Front Surg. 2021;8:681028. doi: 10.3389/fsurg.2021.681028 34422892 PMC8377277

[6] Kim JS . 9-Point injection technique for lip augmentation and lip corner lifting using sonographic imaging of the labial artery pathway. Aesthet Surg J. 2024;44:1080–1090. doi: 10.1093/asj/sjae086 38649792 PMC11483566

[7] Müller DS, Grablowitz D, Krames-Juerss A, Worseg A. Lip augmentation with saypha LIPS lidocaine: a postmarket, prospective, open-label, randomized clinical study to evaluate its efficacy and short- and long-term safety. Aesthet Surg J. 2025;45:84–97. doi: 10.1093/asj/sjae149 PMC11634382 39167667

[8] Nikolis A, Bertucci V, Solish N, Lane V, Nogueira A. An objective, quantitative assessment of flexible hyaluronic acid fillers in lip and perioral enhancement. Dermatol Surg. 2021;47:e168–e173. doi: 10.1097/DSS.0000000000002917 33481441 PMC8078114

[9] Beer K, Glogau RG, Dover JS, et al A randomized, evaluator-blinded, controlled study of effectiveness and safety of small particle hyaluronic acid plus lidocaine for lip augmentation and perioral rhytides. Dermatol Surg. 2015;41:S127–S136. doi: 10.1097/DSS.0000000000000199 25828037

[10] Raspaldo H, Chantrey J, Belhaouari L, et al Aumento de labios y zona perioral: un estudio prospectivo, aleatorizado y controlado de 12 meses de duración. J Drugs Dermatol. 2025;14:1444–1452. doi: 10.36849/JDD.2015.26659938 26659938

[11] Smith SR, Vander Ploeg HM, Sanstead M, Albright CD, Theisen MJ, Lin X. Functional safety assessments used in a randomized controlled study of small gel particle hyaluronic acid for lip augmentation. Dermatol Surg. 2015;41:S137–S142. doi: 10.1097/DSS.0000000000000164 25828038

[12] Weiss R, Beer K, Cox SE, et al A randomized, controlled, evaluator-blinded, multi-center study of hyaluronic acid filler effectiveness and safety in lip fullness augmentation. Dermatol Surg. 2021;47:527–532. doi: 10.1097/DSS.0000000000002856 33587369 PMC8021234

[13] Bertossi D, Nocini R, van der Lei B, et al Lip reshaping with LOVE approach: a prospective analysis based on two hyaluronic acid fillers. Plast Reconstr Surg Glob Open. 2021;9:e3957. doi: 10.1097/GOX.0000000000003957 34849320 PMC8613371

[14] Coppini M, Caponio VCA, Mauceri R, et al Aesthetic lip filler augmentation is not free of adverse reactions: lack of evidence-based practice from a systematic review. Front Oral Health. 2024;5:1495012. doi: 10.3389/froh.2024.1495012 39483115 PMC11525007

[15] American Society of Plastic Surgeons . ASPS Evidence Rating Scales: Evidence Rating Scale for Therapeutic Studies. 2011. Accessed January 8, 2026. https://www.plasticsurgery.org/documents/medical-professionals/health-policy/evidence-practice/ASPS-Rating-Scale-March-2011.pdf

[16] Buhsem O . Comparing the effects of different injection techniques used in lip augmentation on filler migration and patient satisfaction. Cureus. 2024;16:e64716. doi: 10.7759/cureus.64716 39021739 PMC11253074

[17] Hilton S, Frank K, Alfertshofer M, Cotofana S. Clinical outcomes after lip injection procedures—comparison of two hyaluronic acid gel fillers with different product properties. J Cosmet Dermatol. 2023;22:119–127. doi: 10.1111/jocd.15548 36459413

