# Supplementary material for: Hyaluronic Acid Injection Techniques for Lip Augmentation—Comparison of Linear, Retrograde, and Microdeposit Approaches: A Systematic Review
Source: Aesthet Surg J Open Forum. 2026 Apr 2;8:ojag059. doi: 10.1093/asjof/ojag059 (PMC13108440; doi:10.1093/asjof/ojag059)
Supplement: ojag059_Supplementary_Data [file ojag059_supplementary_data.docx]

**Supplemental Table 1**. Characteristics of Included Studies Evaluating Hyaluronic Acid Injection for Lip Augmentation

| Study | Population | Injection technique & instrument (gauge/length), as reported | Procedure Type | HA product | Volume | Satisfaction | Study Period (recruitment)/Follow-up (as reported) | Complications | Study Type | Rheology (G'/viscosity/cohesivity; cross-linking) |
| --- | --- | --- | --- | --- | --- | --- | --- | --- | --- | --- |
| Keramidas E, Rodopoulou S. (1) | 833 | Needle (NR);  Technique:  (Linear Retrograde). | Injecting the upper lip followed by the lower lip. | Brand not specified. (25 mg/mL). | 1 a 1.5 mL for both lips. | GAIS (5-point) + patient questionnaire; assessed immediately post-procedure and Day 15. | 2013 – 2019 (study period). | Severe Hematomas (8) and Severe Inflammation (10). | Clinical Trial | Cross-linking 3/6; concentration 25 mg/g (No G’/G’’/viscous). |
| Rho NK, Goo BL, Youn SJ, Won CH, Han KH. (2) | 36 | Needle: 27G;  Technique: (Retrograde technique). | Group 1 and 2: injecting the upper lip followed by the lower lip. | Group 1: L’ORIENT No2 Group 2: L’ ORIENT No4  (20 mg/mL) + Lidocaine 0.3%. | 1 mL in the lips (0.4 mL in the upper and 0.6 mL in the lower lip). | Study-specific satisfaction scale (0-3; not satisfied to very satisfied) for volume, shape, attractiveness, and naturalness. | 12 weeks (follow-up). | Group 1: Local swelling (1).  Group 2: Hematoma (2) were temporary. | Randomized Single-Blinded Study | BDDE; Degree of cross-linking 1-2% vs 2-3%; Elastic Modulus G’ 249 vs 436 Pa; Viscous modulus G’’ 34.67 vs 43.70 Pa; Tan 𝛿 0.14 vs 0.10; Cohesiveness -0.21 N (both). |
| Hilton S, Sattler G. (3) | 60 | Needle: 30G (½ inch).  Technique:  (Linear Retrograde/Anterograde threading, serial puncture, and fan or fern pattern for lip injection). | Injecting the upper lip followed by the lower lip. | Restylane Kysse (RK) (20 mg/mL) vs AH JV (15 mg/mL) + Lidocaine. | Inject up to 3 mL (1.5 mL in each lip). | Patient satisfaction: 96% with AH RK and 91% with AH JV at 12 months (as reported). | 12 months (follow-up). | Pain (4 for AH RK and 1 AH JV)  Inflammation (2 for AH RK and 1 AH JV) .  No EAs at 12 months. | Randomized Clinical Trial | No rheology/physicochemical metrics reported. |
| Gonzalez C, Callejas E. (4) | 27 | Needle (NR); Cannula: 25G x 50 mm.  (11 patients with Needle and 16 with cannula). Technique:  Linear Retrograde. | Needle: From lateral to medial.  Cannula: From lateral to medial. | Restylane Kysse (RK). | 0.4 – 1 mL for both lips. | Study-specific subjective satisfaction scale (1-5): 100% satisfied; 74% “complete satisfaction” (score = 5). | 8 months (follow-up). | No complication. | Cohort Study | Elasticity 156 Pa; high cohesivity (qualitative). (No G’/G’’/Tan ∂). |
| Czumbel LM, Farkasdi S, Gede N. (5) | 1,228 | Various injection technique (NR). | Various injection technique. | Juvéderm and Restylane and others . | Various injection volumes. | NR (meta-analysis/systematic review; satisfaction outcomes not extractable by technique). | 12 months (follow-up). | Pain on palpation: 88.8%; hematomas: 39.5%; Granuloma: 0.6%; Herpes Labialis: 0.6%. | Meta-analysis and Systematic Review | No rheology/physicochemical metrics reported. |
| Kim JS. (6) | 50 | Needle (NR); Technique: (Linear threads in an anterograde-retrograde technique). | Injecting the upper lip followed by the lower lip. | Yvoire Classic Plus (20 mg/mL) + Lidocaine. | 1 mL for both lips. | Patient-rated GAIS (-1 to +3), assessed at 1 week, 1 month, and 3 months (mean GAIS: 2.8, 2.4, and 1.9, respectively). | 2017 – 2022 (study period). | No major complication. | Cohort Study | No rheology/physicochemical metrics reported. |
| Müller DS, Grablowitz D, Krames-Juerss A (7) | 114 | Needle: 27G (½ inch); Cannula: 25G x 50 mm; Cannula: 22G x 70 mm.  Technique:  (Linear Retrograde, Bolus technique with needle and cannula). | Group 1: Retrograde  Group 2: Bolus  Group 3: Needle  Group 4: Cannula. | Saypha LIPS (23 mg/mL) Lidocaine. | 0.79 mL for upper lip and 0.8 mL for lower lip. | FACE-Q + GAIS (patient and investigator) + patient satisfaction questionnaire; assessed at Week 6 and follow-up at Months 6, 12, and 18. | 18 months (follow-up). | Overall, AE rate by technique (no event-type breakdown reported): retrograde 77.5%; Bolus 86.1%. (as reported). | Randomized Controlled Trial | Cross-linked with BDDE; G’ (elastic modulus) ~ 150,000 mPa (1 rad/s); viscous modulus ~ 27,000 mPa (as reported). |
| Nikolis A, Bertucci V, Solish N, Lane V. (8) | 59 | Needle and Cannula (NR); Technique:  (Serial Puncture, linear threading, and/or crosshatching). | Injecting the upper lip followed by the lower lip. | Restylane Kysse (RK) vs AH rk + AH rr/ AH rd. | 1.80 mL for both lips. | NR | 2 months (follow-up). | NR (timing/complications not specified). | Prospective Clinical Trial | No rheology/physicochemical metrics reported. |
| Beer K, Glogau RG, Dover JS, Shamban A. (9) | 199 | Needle (NR); Technique: (Linear retrograde-anterograde threading, and serial puncture). | Injecting the upper lip followed by the lower lip. | Restylane SGP (20 mg/mL). | 1.5 mL for both lips. | GAIS (7-point; patient-and investigator-rated), assessed at Weeks 2, 4, 8, 12, 16, 20, and 24. | 9 months (follow-up). | 6 patients (2%) reported severe TRAEs “began within 1 day” and “resolved within 6-10 days" (lip swelling). | Randomized Clinical Trial | Degree of cross-linking mentioned; “rheological properties” discussed (qualitative); no numeric G’/G’’/viscosity/cohesivity). |
| Raspaldo MD,  Jonquille Chantrey MD (10) | 280 | Needle: 30G (½ inch);  Technique:  (Retrograde technique 75%) (Anterograde technique 26.7%). | Injecting the upper lip followed by the lower lip. | Juvederm Volbella + Lidocaine vs Restylane-L. | 1.2 mL (range 0.5 – 2 mL). | FACE-Q modules: Recovery Early Life Impact and Recovery Early Symptoms and Satisfaction with Lips and Satisfaction with Outcome | 12 months (follow-up) | Injection-site responses recorded; most ≤14 days; subset 15-30 days. | Randomized Controlled Trial | No rheology/physicochemical metrics reported. |
| Smith SR, Vander Ploeg HM (11) | 180 | Needle: 30G (½ inch);  Technique:  (Linear Retrograde and Anterograde technique). | Injecting the upper lip followed by the lower lip. | Restylane (20 mg/mL). | ≤1.5 mL upper lip and ≤1.5 mL lower lip. | GAIS: 99% rated “improved or better”; 77% chose retreatment (as reported). | 6 months (follow-up). | Lip asymmetry  (Mild: 7.4%; Moderate: 0.5%; and severe 0.4%). | Randomized Clinical Trial | “Viscoelastic properties” described qualitatively; no numeric rheology reported. |
| Weiss R, Beer K, Cox SE, Palm M, Kaufman-Janette J. (12) | 280 | Needle (NR);  Technique: (Linear Retrograde/Anterograde threading, serial puncture, and fan or fern pattern for lip injection). | Injecting the upper lip followed by the lower lip. | Restylane Kysse (RK) vs Juvederm Volbella (JV). | NR (volume reported as total treatment volume; lip-specific volume not extractable). | FACE-Q (Rasch-transformed total scores for satisfaction with lip and appraisal of lip lines). | 12 months (follow-up). | Lumps (RK; 10% vs JV; 11%) and hematoma (RK; 8% vs JV; 10%) lasted less than 7 days. | Randomized Clinical Trial | No rheology/physicochemical metrics reported. |
| Bertossi D, Nocini R, van der Lei B, Magistretti P. (13) | 60 | Needle and Cannula (NR); Technique: (Linear Retrograde and Bolus). | Group 1: 20 patients aged between 20 and 34 years. Group 2: 20 patients aged between 35 and 45 years. Group 3: 20 patients aged ≥46 years. | Vycross (Volift and Volite). | Group 1: 1.1 ± 0.1 mL Group 2: 1.5 ± 0.1 mL  Group 3: 1.6 ± 0.2 mL. | Likert Score (0 – 6), by age group: 20-34y 4.9/6; 35-45y 4.8/6; ≥46y 4.8/6 (as reported). | 6 months (follow-up). | Edema: 88%  Hematomas: 17%; no major complications. | Cohort Study | VYC-17.5: intermediate G’ (qualitative), HA 17.5 mg/mL; VYC-12: very low G’ (qualitative), HA 12 mg/mL. |
| Coppini M, Caponio VCA, Mauceri R, Pizzo G, Mauceri N, Lo Muzio L, et al. (14) | 30 | NR | Injecting the upper lip followed by the lower lip. | NR | NR | NR (systematic review focused on adverse reactions; patient satisfaction outcomes not extractable. | 24 months (follow-up). | Granulomatous foreign body reaction 76.6% (24 months). | Systematic Review | No rheology/physicochemical metrics reported. |
| Buhsem O. (16) | 216 | Needle 30G; Cannula 23G;  Technique: (Linear technique). | Group 1: from top to bottom.  Group 2: from bottom to top.  Group 3: From lateral to medial  Group 4: From lateral to medial (cannula). | YVOIRE Volume Plus (20 mg/mL) + Lidocaine 0.3%. | 0.6 mL in the upper lip. | Study-specific 5-point satisfaction scale (0-5), by injection-direction group: G1 4.78/5; G2 3.70/5; G3 4.15/5; G4 3.85/5 (as reported) | 2017 – 2023 (study period). | No complications were found. | Randomized Controlled Trial | No rheology/physicochemical metrics reported. |
| Hilton S, Frank K, Alfertshofer M. (17) | 40 | Needle (NR);  Technique:  (Linear Retrograde). | Injecting the upper lip followed by the lower lip. | Restylane Kysse (RK) (20 mg/mL)  Vs JUS (24 mg/mL). | 1 mL for both lips. | Subject satisfaction questionnaire + subject-assessed GAIS; assessed at baseline/screening, Day 0, Day 14, and Weeks 4, 12, 24. | 6 months (follow-up). | Moderate Severe (2) (AH JUS)  Moderate erythema (2) (AH JUS) Sensitivity/pain (8) (AH RK). | Controlled Clinical Trial | No rheology/physicochemical metrics reported. |

HA, hyaluronic acid; n, number of patients; GAIS, Global Aesthetic Improvement Scale; FACE-Q, FACE-Q patient-reported outcome measure; VAS, visual analog scale; AEs, adverse events; NR, not reported. Rheologic/physicochemical properties were recorded only when explicitly stated in the article and were not inferred; RCT, randomized controlled trial Percentages of injection instruments and techniques reported in the results section are calculated only from studies that explicitly provided data for that variable.
